# Typhoid Fever

**Published:** 1880-12-20

**Authors:** J. H. Egan

**Affiliations:** Pulaski, Tenn.


### Southern Medical Record.

EDITORS:

T. S. POWELL, M.D. W. T. GOLDSMITH, M.D. R. C. WORD, M.D.

*R. C. WORD, M.D., Managing Editor.*

☞ All Communications and Letters on Business connected with the RECORD must be addressed to the Managing Editor.

VOL. X. ATLANTA, GA., DECEMBER 20, 1880. NO. 12.

### ORIGINAL AND SELECTED ARTICLES.

### TYPHOID FEVER.

BY J. H. EGAN, M.D., OF PULASKI, TENN.

Until the present century great confusion existed as to typhus and typhoid fevers. Both diseases were described under the same name and considered identical. Typhus fever was unknown on the continent of Europe and America, and the description given of that fever appertained to typhoid. Typhus was the endemic fever of Great Britain, and occasionally there were outbreaks of typhoid. When the immense tide of emigration flowed into the United States it brought along with it the typhus of the mother country. Ship fever, which has been most graphically described, is typhus. The non-identity of typhus and typhoid was repeatedly asserted from 1830 to 1840; but to Sir William Jenner is due the credit of recognizing the specific differences between the two diseases. Since the writer's first visit to the United States a large number of physicians believed in the identity of the two diseases. The profession in the United States are under obligations to Dr. William Bartlett for tabulating the differences in symptoms between the two fevers; and after perusing his work no candid person could fail to admit that they are separate and distinct. There could be no excuse for a physician educated in Great Britain in late years failing to recognize the non-identity of these fevers, as he had an opportunity of seeing hundreds of both; but it was otherwise in Europe and the United States, as typhus was a rare disease. Dr. Geo. B. Wood gave the name of enteric fever to typhoid, which was an excellent name, and pointed to its invariable pathological lesion. Typhoid had previously been given to it, and it is difficult to change a name which has come into common use. A worse name could not well have been given, as it gives rise to constant confusion. We have typhoid fever and the typhoid state, which is incidental to many diseases, and when attached to the name of another disease, as typho-malarial fever or typhoid pneumonia, gives rise to endless confusion. In the seventeenth century we find descriptions of cases and autopsies showing that typhoid fever was widely spread in Europe; and in the eighteenth century the existence of the disease is beyond doubt. The writings of Willis Sydenham, Hoffman and Morgagni attest the matter. Typhoid fever is infectious to a slight degree; but this infection does not extend beyond a few feet from the patient. Defective sewerage, contaminated milk and water containing the disease germs, are the chief causes of typhoid fever. The fever was unknown in Queensland, Australia, until 1864, when the American ship "Flying Cloud" carried it there in the water which it had on board. Liebermeister, in his article on typhoid fever in Ziemssen Clyclopædia, enounces the doctrine that the poison of typhoid fever does not originate in decomposing substances, but finds in them favorable ground for growth and multiplication. He says "there are multitudes of houses in which the effluvia of the privies can be smelt through all the rooms, and in which the inhabitants are constantly inhaling sewer gases; and neither the temporary nor permanent residents are attacked with typhoid fever. Cities with defective sewerage are not by any means always visited with typhoid fever." Dr. Budd established the doctrine that the poison of typhoid fever is propagated continuously. It follows, then, that if a single case be introduced into a community, it may give rise to single cases or epidemics. The transporters of the poison is the excrement, and it is the washing of this into the wells which contaminates the water and produces the disease. Every case of typhoid fever can be traced to a specific source, if energetically sought for. The poison of typhoid fever retains its vitality for a longer time than that of any other disease with which we are acquainted.

The period of incubation is difficult to determine, but is supposed to be from fourteen days and upwards. The longest known period is twenty-eight days. The average is twenty-one days. The mode in which the poison gains entrance to the system is either by the air we breathe or the water we drink. This is the reason why the washerwomen, who wash the bed-linen soiled by the dejections of the patients, are more frequently attacked than the other attendants.

The primary local lesions of typhoid fever, and one which is always present in every case of the disease, and is pathognomonic of it, is ulceration of Peyer's patches and the solitary glands, and also the changes in the mesenteric glands and in the spleen. There are other lesions which vary in different cases, and are the results of the general disease; such are the degeneration of the liver, kidney and heart; but these depend upon the intensity and duration of the general diseases and the severe and long-continued fever.

Typhoid fever develops very slowly, so much so that patients cannot tell when they began to get sick. It is usually from four to ten days before it is necessary to take to the bed. This is characteristic of the dlsease. A slight bronchitis accompanies typhoid fever, together with epistaxis, and this generally occurs in the early part of the fever. Deafness, either slight or almost complete, frequently occurs. The bowels are loose and dejections of a yellowish color, and in connection therewith is tenderness of the illiac region on the right side. The abdomen is tympanitic, owing to the accumulation of gas in the intestines.

Griesenger says: "The fever in a measure controls the situation." The fever lasts, as a rule, from three to four weeks. It is a self-limited disease, but these limits are often very wide. Fourteen days is the shortest duration, and there have been cases to last ninety days. The general duration, however, is four weeks. In the first week there is a gradual and steady increase of the fever; in the second week the fever is continuous; in the third week the fever begins to be remittent; but in the exascerbations the highest temperature is reached; in the fourth week the fever becomes intermittent, and the exascerbations become lower.

The disturbances of the nervous system depend upon the intensity of the fever. There may be general malaise, restlessness, headache and unquiet sleep; or there may be slight disturbance of the intellect; or there may be a low muttering delerium, a drowsy condition from which the patient can be temporarily aroused; or the delerium may be violent; or there may be constant loss of consciousness from which the patient cannot be aroused. If the case be severe, there will be subsultus tendinum, tinnitus aurium and extreme nervousness.

On the seventh day of the disease there will generally be observed a slight roseolar eruption, which disappears on pressure. This is not always present.

Pneumonia, pleurisy, erysipelas and organic disease of the heart frequently are complications of typhoid fever.

Relapses are not uncommon in typhoid fever. When they do occur there is a sudden bound up in the temperature which does not attain its height gradually, as in the first attack, but remains high for a short time, and then we have the morning and evening changes. The morning remissions and evening exascerbations are much more marked in the relapse than in the original attack. The eruption makes its appearance with the first symptoms of the relapse, and continues until the fever disappears. The spleen never decreases in size while the fever remains, and in a relapse swells up again.

The treatment of typhoid fever has undergone a great change in the United States since the writer first visited the New York hospitals. Dr. Alonzo Clark was the first to introduce the Edinburgh or conservative plan of treatment in Bellevue Hospital. Fires were lighted in the wards to produce a current of air; the windows were thrown open; water was given ad libitum, and the patients were kept as cool as possible. To use his own expression: "Under the old treatment the patients went out in coffins, and under the new on their own limbs." I have seen three hundred patients in the Edinburgh Infirmary, not one of whom got a teaspoonful of medicine—only food and stimulants. This was carrying matters to the other extreme, as I am certain that treatment directed to the prominent symptoms would have been beneficial.

The prominent symptom is the fever. The temperature must never be allowed to exceed 103° Fah.; as soon as this point is reached measures must be taken to reduce it. This can be done in two ways, either by cold water or by medicine. I prefer the former; although I am free to confess that I have seen the most marvellous results from the use of large doses of quinine. In the practice of Dr. Compton, of Evansville, Indiana, I saw exhibited two doses of twenty grains, one hour apart, with the result of bringing the temperature down at once two degrees, and keeping it there. Water can be employed in the cold bath—cold pack and sponging; the latter I prefer, when it will accomplish the end, but this it will not always do. The whole body may be sponged with water at a temperature which is agreeable to the patient, and a small portion of the surface be denuded and sponged until the application has been made over the whole person. There is no danger in the cold pack sheet, provided all below the knees be left uncovered. There is not much benefit to be derived from it, and the good effects are not derived from the cold. The cold bath is a troublesome method, and frequently impracticable to give in a private house, and is liable to produce some nervous shock, and on this account is not often used. The same results can be obtained by simply wrapping the patient in a wet sheet and sprinkling with cold water. Personally, I use sponging with cold water, and if this be ineffectual, then I prefer the "Kibbee"

water bed. This was used in Memphis in the epidemic of yellow fever in 1873, and subsequently in 1878 at New Orleans, where the inventor lost his life by yellow fever. Dr. T. G. Thomas has used it to reduce the temperature in cases of ovariotomy, and speaks of it highly. Whatever be the plan adopted, an effort must be made to reduce the temperature to 100° Fah.

The amount of fluid required for the healthy performance of function is 120 ounces per diem. We may safely say that not less than two pints ought to be given daily to the patient. If the intellect be clear, the desires of the patient will be the best indication for the amount of the fluid, and the frequency it ought to be given. When the intellect is clouded and the mouth dry, a tablespoonful ought to be given frequently. It will be found that on presentation of spoon to the lips the patients will mechanically swallow what is offered.

I have long been in the habit of administering a febrifuge which, if it does no good, will certainly do no harm. In some cases it seemed to be advantageous. I use sulphurous acid, largely diluted with water, three drachms to a pint of water; in place of two ounces of water, the same amount of syrup can be added.

To guard against the gravitation of the blood into the posterior aspect of the lungs, which is one cause of the œdema and pneumonia complicating this fever, it is a good plan to change the position of the patient and not permit him to lie on his back all the time. It will also prevent the formation of bed-sores, which adds to the nervousness of the patient and drains his strength.

Should the discharge from the bowels consist of small semi-solid stools, it ought to be disregarded. Nothing is so detrimental to a patient as over-medication. If, however, the stools be watery and large, showing follicular intestinal catarrh, opium and astringents must be used. Nitrate of silver is a very popular astringent with some. If the bowels be constipated, no cathartic is to be exhibited, but minute doses of belladonna, which will induce an operation.

For the tympanites, turpentine, in the form of an emulsion, is the best remedy. The prescription which I administer is:

℞ Ol. terebinthæ.

Liq. potassæ, a.a.......................................... ʒ ii.

Mucil acaciæ.............................................. ʒ iv.

Syrup papav alb.:

Syrup floris aurantii, a.a.................................. ℥ i.

Aquæ camphoræ, a.d....................................... ℥ viii.

M. Sig.: A tablespoonful every four hours.

If the tympanites be excessive and not mitigated by the above, it will be necessary to pass a rubber tube up intestine and allow the gas to escape.

For the nervousness, if excessive, we have found nothing to equal fl. ex. lupulin in small doses, or Hosford's acid phosphate.

In reference to diet, we are opposed to solid food during the continuance of the fever, and for sometime thereafter. I know of nothing which has given me so much satisfaction as a milk diet, and to this may be added, when there is failure of the vital powers, sanguis bonvinus exsicatus. In the third week it often becomes necessary to administer a stimulant. I never give whisky—simply Hungarian wine or the finest French brandy.

Typhoid fever cannot be cured by medicine. In a large proportion of cases nothing is requisite but rest in bed, quietude, fresh air, pure water and a regulated diet. When medicaments are given to hold special symptoms in check, it is better at once to discontinue them as soon as the indication has been met.

